# Enhanced Microvascular Adaptation to Acute Physical Stress and Reduced Oxidative Stress in Male Athletes Who Consumed Chicken Eggs Enriched with n-3 Polyunsaturated Fatty Acids and Antioxidants—Randomized Clinical Trial

**DOI:** 10.3390/life13112140

**Published:** 2023-10-31

**Authors:** Luka Kolar, Petar Šušnjara, Marko Stupin, Ana Stupin, Ivana Jukić, Zrinka Mihaljević, Nikolina Kolobarić, Iva Bebek, Diana Nejašmić, Marija Lovrić, Ines Drenjančević

**Affiliations:** 1 Department of Internal Medicine, National Memorial Hospital Vukovar, 32000 Vukovar, Croatia; lukakolar.vu@gmail.com; 2 Scientific Center of Excellence for Personalized Health Care, Josip Juraj Strossmayer University of Osijek, 31000 Osijek, Croatia; psusnjara@mefos.hr (P.Š.); ana.stupin@mefos.hr (A.S.); ivana.jukic@mefos.hr (I.J.); zmihaljevic@mefos.hr (Z.M.); nikolina.bilic.dujmusic@gmail.com (N.K.); 3 Department of Physiology and Immunology, Faculty of Medicine Osijek, Josip Juraj Strossmayer University of Osijek, 31000 Osijek, Croatia; 4 Department for Cardiovascular Disease, University Hospital Centre Osijek, 31000 Osijek, Croatia; 5 BICRO BIOCENTAR d.o.o., 10000 Zagreb, Croatia; ivabebek@gmail.com (I.B.); diana.nejasmic@biocentre.hr (D.N.); marija.lovric@biocentre.hr (M.L.)

**Keywords:** athletes, enriched eggs, functional food, microcirculation, oxidative stress

## Abstract

This randomized interventional study aimed to determine the effects of n-3 polyunsaturated fatty acids, selenium, vitamin E, and lutein supplementation in the form of enriched chicken egg consumption on microvascular endothelium-dependent vasodilation, oxidative stress, and microvascular response to an acute strenuous training session (ASTS) in competitive athletes. Thirty-one male athletes were assigned to a control (n = 17) or a Nutri4 group (n = 14) who consumed three regular or enriched chicken eggs per day, respectively, for 3 weeks. Significantly enhanced endothelium-dependent responses to vascular occlusion (PORH) and iontophoresis of acetylcholine (AChID) were observed in the Nutri4 group but not in the control group after egg consumption. Formation of peroxynitrite and hydrogen peroxide in peripheral blood mononuclear cells, as well as serum concentration of 8-iso prostaglandin F2α, decreased in the Nutri4 group while remaining unchanged in controls. PORH and AChID were reduced post-ASTS compared with pre-ASTS, both before and after the diets, in both groups. However, the range of PORH responsiveness to ASTS (ΔPORH) increased after consumption of enriched eggs. These results suggest that consumption of enriched chicken eggs has a beneficial effect on microvascular endothelium-dependent vasodilation and the reduction of oxidative stress levels in competitive athletes. Also, microvascular adaptation to the ASTS was improved after consumption of Nutri4 eggs.

## 1. Introduction

Proper nutrition has a very important role in the performance of professional athletes, helping them to maintain ideal body weight and body composition specific to sports and faster recovery [1]. However, it is alarming that nutritional practices of elite athletes are often suboptimal, considering that the pursuit of better results and new records is forcing athletes to train longer and harder than ever. Analyses of dietary habits in various athlete groups found that a substantial proportion of the studied populations did not reach the dietary goals for many macro- and micronutrients, including n-3 polyunsaturated fatty acids (n-3 PUFAs), vitamins, and trace elements such as iron (Fe), zinc (Zn), and selenium (Se) [2,3]. Proper intake of micronutrients, especially those with antioxidant properties, is important to maintain oxidative balance and physiological homeostasis in athletes since strenuous exercise and overtraining are known to increase acute oxidative stress [4,5]. Increased oxidative stress and reduced antioxidant capacity (measured as reduced ferric reducing ability of plasma (FRAP), increased 8-iso-prostaglandin F2α level (indicator of in vivo lipid peroxidation), increased total oxidant status [6], increased production capability of reactive oxygen species (ROS) in peripheral blood mononuclear cells (PBMCs) [7], etc.), have been repeatedly demonstrated following exercise training. Today, athletes are also very interested in nutrients that can improve athletic performance and recovery; they often use them to increase metabolic capacity, improve muscle growth, delay the onset of fatigue, and shorten recovery times [8]. However, nowadays, athletes avoid taking large amounts of supplements, especially in the form of pills [9]. Thus, to achieve better sports performance but also a healthier and more natural diet, consuming so-called functional foods could have a two-fold potential in the nutrition of elite athletes. The term “functional foods” refers to foods of natural origin that, in addition to an adequate nutritional effect, have a beneficial effect on one or more target functions in the body by improving health and general well-being or reducing the risk of disease [10].

Recently, a new functional food has been developed, namely, chicken eggs enriched with n-3 PUFAs, Se, lutein, and vitamin E [11], that could present a means to deliver nutrients in higher concentration per meal in a more convenient way. Chicken eggs are suitable for modification of the fatty acid profile since the content of n-3 PUFAs can be increased with the addition of different proportions of fish oil to the mixtures for laying hens. Eggs are also suitable for the deposition of higher concentrations of antioxidants like Se, vitamin E, and lutein by adding organic Se, marigold extract as a source of lutein, and a vitamin mixture to feed for laying hens, respectively [11]. Because of the anti-inflammatory and antioxidative proprieties of n-3 PUFAs [12], there is an increasing interest in the potential benefits of n-3 PUFA supplementation in athletes. One of the potential antioxidant effects of n-3 PUFAs is the reduction of intracellular oxidative stress, which was demonstrated by the potential of n-3 PUFAs (EPA and DHA) to reduce intracellular H_2_O_2_-induced DNA damage in human aortic endothelial cells [13]. Available data on n-3 PUFA supplementation indicate various improvements in skeletal muscle performance, energy metabolism and endurance performance in a population of athletes. For example, supplementation of n-3 PUFAs for 4 weeks may successfully attenuate minor aspects of exercise-induced muscle damage, e.g., soreness-associated exercise avoidance [14]. On the other hand, even though n-3 PUFA supplementation in healthy volunteers did not affect brachial artery diameter, conductance, and blood flow at rest, the increases in these measures were greater during contraction with the n-3 PUFA supplementation, suggesting the potential of n-3 PUFAs to improve vascular adaptation to exercise [15]. Similarly, a recent study by our research group showed that supplementation with n-3 PUFAs in the form of n-3 PUFA-enriched chicken eggs improved resting microvascular endothelial function and contributed to vascular adaptation to acute exhaustion in competitive athletes [16]. Besides n-3 PUFAs, micronutrients with antioxidant and anti-inflammatory properties have the potential to improve cardiovascular health, especially in conditions characterized by increased oxidative stress [17,18]. Preservation of endothelial vascular function has been shown as a significant protective effect of vitamin E supplementation in both healthy and cardiovascular disease populations, where it manifested as a reduction in carotid intima-media thickness and a reduction in the production of biomarkers of endothelial activation and endothelium–leukocyte interaction [19,20]. Exercise-induced oxidative damage in all endurance athletes and enhanced production of oxidized low-density lipoprotein (oxLDL) following strenuous endurance exercise may be prevented by vitamin E supplementation and by maintaining higher vitamin E status [21]. Because Se is an integral component of several antioxidant enzymes, most notably glutathione peroxidase (GPx), which exerts its antioxidant effects by reducing the concentration of peroxides such as hydrogen peroxide (H_2_O_2_), consumption of Se could play a central role in preventing and influencing the clinical course of various diseases (e.g., cancer, diabetes, cardiovascular disease, infections) [22,23] as well as being used to improve athletic performance and exercise recovery [24]. A recent systematic review reported that Se supplementation did not benefit athletic performance but prevented Se deficiency in athletes with high-intensity training. Optimal plasma Se levels have been shown to be important in minimizing chronic exercise-induced oxidative effects and modulating the training effect on mitochondrial changes [24].

A recent study by our group reported that the serum level of pro-inflammatory cytokine IL -17A decreases and neuronal nitric oxide synthase (nNOS) expression increases after consumption of chicken eggs enriched with n-3 PUFAs, Se, vitamin E, and lutein in young healthy subjects, suggesting that the combined effect of n-3 PUFAs and antioxidant micronutrients may play an important role in improving vascular endothelial properties (particularly those dependent on nitric oxide) and maintaining an inflammation-free environment under resting conditions [25]. Because of the paucity of data on the concomitant action of various micronutrients possessing anti-inflammatory and antioxidative properties, especially in the form of functional food, it would be prudent to investigate the effect of functional foods on vascular endothelial function and oxidative status in demanding populations such as competitive athletes. Since the earliest adaptive changes in physiological conditions, as well as the final organ damage in various pathological conditions, occur precisely at the microvascular level, it is essential to evaluate blood flow responses to standardized stimuli, specifically in the microcirculation. Our hypothesis is that competitive athletes may benefit from the consumption of chicken eggs enriched with n-3 PUFAs, Se, lutein, and vitamin E in terms of microvascular endothelial function and a more favorable oxidative environment. Thus, the present study aimed to (a) investigate the effects of intake of chicken eggs enriched in n-3 PUFAs, Se, lutein, and vitamin E (Nutri4 eggs) on skin microvascular endothelium-dependent and -independent vasodilation in athletes; (b) examine if functional food consumption modifies microvascular adaptation to acute exhausting training; and (c) determine the effects of functional food consumption on oxidative stress and antioxidant capacity in competitive athletes.

## 2. Materials and Methods

### 2.1. Study Population

Thirty-one young, healthy male competitive athletes participated in this study. They were recruited from local rowing and track and field clubs. All participating athletes had been training for at least 1 year, 5 and 12 times per week. In addition to being active competitive athletes, eligibility criteria included age between 18 and 30 years, normal body mass index (BMI, 18.5 to 24.9 kg/m^2^), normal arterial blood pressure values (BP, <140/90 mmHg), and normal serum lipid levels (total cholesterol < 5.00 mmol/L, triglycerides < 1.70 mmol/L, HDL cholesterol > 1.00 mmol/L, LDL cholesterol < 3.00 mmol/L). Exclusion criteria were hypertension, coronary artery disease, diabetes, hyperlipidemia, renal insufficiency, cerebrovascular and peripheral artery disease, history of smoking, and use of drugs or substances that might affect the endothelium. Written informed consent was obtained from each subject. The study protocol and procedures met the standards of the latest revision of the Declaration of Helsinki and were approved by the Ethics Committee of the Faculty of Medicine, University of Osijek, Osijek, Croatia (Cl: 602-04/21-08/07; No: 2158-61-07-21-151). This study is part of a clinical research study investigating the effects of functionally enriched chicken eggs on cardiovascular function registered at ClinicalTrials.gov (accessed on 11 September 2023) (NCT04564690).

### 2.2. Production of n-3 PUFA-, Selenium-, Lutein- and Vitamin E-Enriched Chicken Eggs

Chicken eggs enriched with n-3 PUFAs, lutein, Se, and vitamin E were prepared according to the protocol of a research group from the Faculty of Agrobiotechnical Sciences, University of Osijek [11]. Rapeseed oil (1.5%) in the feed mixtures fed to laying hens was replaced by a mixture of fish oil (1.5%) and linseed oil (2%), with 0.43 mg/kg of selenium mixture and 100 mg/kg of mixture of vitamin E and lutein added. Such feed mixture for laying hens resulted in the production of Nutri4 eggs, the content of which is described in detail in the study of our collaborators from the Faculty of Agrobiotechnical Sciences Osijek [11].

### 2.3. Study Design

Subjects were instructed to eat three hard-boiled chicken eggs daily (63 eggs total) during the 21-day study protocol. Participants were divided into the experimental Nutri4 group (14 subjects), which consumed chicken eggs fortified with n-3 PUFAs, Se, lutein, and vitamin E (three per day; approximately 1056 mg n-3 PUFAs per day, 0.0573 mg Se per day, 3.29 mg vitamin E per day, and 1.85 mg lutein per day) and in the control group (17 subjects) who consumed normal chicken eggs from the same farm (three per day; about 438 mg n-3 PUFAs per day, 0.0549 mg Se per day, 1.785 mg vitamin E per day, and 0.33 mg lutein per day). Regular and Nutri4 chicken eggs were the same size (commercial size M), and neither the subject nor the researcher knew which group the subjects belonged to. For the purposes of the study, subjects were instructed not to consume any other foods rich in n-3 PUFAs, Se, lutein, and vitamin E, or any other form of these micronutrients during the study protocol, but only the eggs given to them for study purposes. The study was conducted in the Laboratory for Clinical and Sports Physiology, Department of Physiology and Immunology, Faculty of Medicine, University of Osijek. The study consisted of two visits, and all measurements described below were taken on the first day and the day immediately following the end of the protocol. All tests took place in the morning after an overnight fast; participants were instructed not to engage in strenuous activity in the 24 h before the tests.

### 2.4. Basic Anthropometric, Cardiovascular, and Biochemical Measurements

Height and body weight, to calculate subjects’ BMI, were measured using a personal scale with a height meter (RADWAG, Radom, Poland). A tape meter was used for measurement of waist and hip circumference to calculate waist-to-hip ratio (WHR). Three consecutive measurements of arterial BP and heart rate (HR) were performed in the sitting position with an automatic oscillometer (OMRON M3, OMRON Healthcare Inc., Osaka, Japan). Venous blood samples were collected using a blood collection system and vacutainers (BD Becton, Dickinson and Company, Franklin Lakes, NJ, USA). Fasting venous blood samples were analyzed for complete blood count and standard biochemical measurements, including electrolytes (potassium, sodium), urea, creatinine, lipid profile (total cholesterol, triglycerides, LDL cholesterol, and HDL cholesterol), blood glucose, and high-sensitivity C-reactive protein (hsCRP). These standard biochemical analyses were performed at the Department of Clinical Laboratory Diagnostics, Osijek University Hospital, Osijek, Croatia.

### 2.5. Analysis of Fatty Acid Profile, Selenium, Vitamin E, and Lutein Levels in Serum

According to the protocol described in a previous work of our research group, serum samples were analyzed for serum fatty acid profile (37 fatty acids in total) by gas chromatography-tandem mass spectrometry (GC-MS/MS system Thermo Fisher GC Trace 1300 coupled with a TSQ 9000 Triple Quadrupole) in the Bioanalytical Laboratory of BIOCentre, BIOCentre—Incubation Center for biosciences, Zagreb, Croatia.

Serum vitamin E concentration was determined according to a standardized protocol [26], in which serum proteins were first denatured with absolute ethanol, and the supernatant was separated from the proteins with xylene. After separation of the supernatant, 2,2-bipyridyl and FeCl3 were added to the mixture, resulting in pink staining. After 2 min of incubation, absorbance was measured at 492 nm using an ELISA READER. The absorbance obtained was proportional to the vitamin E concentration in serum.

The protocol for measuring Se concentration in serum samples was optimized by the partner institutions in the project [27]. Serum samples were digested in ultrapure HNO_3_ and H_2_O_2_ (5:1 ratio) for 60 min at 180 °C in a CEM Mars 6 closed microwave system (CEM, Matthew, NC, USA). Inductively coupled plasma mass spectrometry (ICP-MS) (ICP-MS, Agilent 7500a Agilent Technologies Inc., Santa Clara, CA, USA) was used to determine the Se concentration in the solution of the digested serum samples. Each serum sample was analyzed by ICP, and the analytical method was controlled by the reference material NIST 1567b (wheat flour, National Institute of Standards and Technology, Gaithersburg, MD, USA).

Determination of lutein concentrations in serum samples was performed according to the existing protocol [28]. In 200 μL of serum, 1 mL of deionized water and 0.01% ascorbic acid dissolved in absolute ethanol were added and stirred in the mixture. Then, 2 mL of hexane was added, stirred, and centrifuged at 2500 RPM for 20 min. After centrifugation, the supernatant was separated and then evaporated, and the lutein concentration was determined by high-performance liquid chromatography (HPLC). HPLC analysis was performed at the Department of Chemistry, Josip Juraj Strossmayer University of Osijek.

### 2.6. Evaluation of Peripheral Microvascular Reactivity

Microvascular reactivity was assessed by laser Doppler flowmetry (LDF) (MoorVMS-LDF, Axminister, UK). Post-occlusive reactive hyperemia (PORH) and acetylcholine iontophoresis (ACh) were performed to assess endothelium-dependent responses, whereas sodium nitroprusside iontophoresis (SNP) was performed for endothelium-independent responses. The PORH assay involved measurement of microvascular blood flow before, during, and after the release of a 1-min vascular occlusion. During baseline flow (B), occlusion (O), and reperfusion (R), microcirculatory blood flow was determined using software that calculated the area under the curve (AUC) for 1-min intervals, and the result was expressed as the difference between the percent change in flow during reperfusion (R% = R/B∗100) and occlusion (O% = O/B∗100) relative to baseline (R-O% increase = R% − O%). In addition, after baseline microvascular blood flow using anode current, either the positively charged ACh (1%) or the negatively charged SNP (1%) was iontophorected as a vasodilator to obtain the stable plateau of maximal LDF response according to adapted established protocols [29]. Software to calculate AUC (1 min intervals) was also used to determine baseline microcirculatory blood flow and during ACh or SNP administration. The result was expressed as the increase in blood flow relative to baseline after administration of ACh or SNP (ACh or SNP blood flow increase).

### 2.7. Protocol for Acute Strenuous Training Sessions

Participants underwent an acute strenuous training session (ASTS) in the form of a rowing protocol to examine the effects of a specific dietary protocol on the adaptation of microvascular function to a specific challenge. The rowing protocol was performed on the Dynamic Indoor Rower Concept 2 rowing ergometer (Concept2 Inc., Morrisville, VT, USA). The ASTS included a progressively incremental rowing protocol modified to include 5 × 4 min submaximal stages and a single maximal stage [29]. The initial workload of the training session was 150 W, with increments of 40 W in the stages. The submaximal stages were separated by 1 min recovery periods, and a 5 min rest was given before the maximal stage, during which subjects were instructed to row at maximal power until exhaustion. For the subjective assessment of physical activity by individuals, we used the category ratio scale (CR10) introduced by Borg, with values ranging from 0 (not at all) to 10 (very strenuous activity). The difference in microvascular reactivity to administration of vascular occlusion (PORH), ACh (AChID), and SNP (SNPID) before and after ASTS indicates the range of microvascular reactivity and is expressed as ΔPORH, ΔAChID, and ΔSNPID. Delta value (Δ) was determined at each study visit before and after completion of each dietary protocol.

### 2.8. Measurement of Biomarkers of Oxidative Stress and Antioxidant Protection

Serum activity and concentration of the enzyme myeloperoxidase (MPO), which catalyzes the formation of various reactive oxygen species (ROS) and is produced mainly by polymorphonuclear neutrophils, were measured. A colorimetric human myeloperoxidase assay kit (ab105136, abcam9) and a commercially available ELISA kit (Human Myeloperoxidase Kit ab272101, Abcam, Cambridge, UK) were used to determine serum MPO activity and concentration, respectively, according to the manufacturer’s instructions.

Serum activity of antioxidant enzymes catalase (CAT), glutathione peroxidase (GPx), and superoxide dismutase (SOD) was tested using the Lambda 25UV-Vis spectrophotometer equipped with the UV WinLab 6.0 software package (PerkinElmer for the Better, Waltham, MA, USA), according to the protocol established in the Laboratory of Biochemistry, Department of Biology, University of Osijek [25].

Serum protein concentration of 8-iso-prostaglandin F2α (8-iso-PGF2α), a product of non-enzymatic peroxidation of arachidonic acid in membrane phospholipids, was measured using a commercially available ELISA kit (Elabscience, catalog number: E-EL-0041) according to the manufacturer’s instructions.

Assessment of intracellular ROS production in peripheral blood mononuclear cells (PBMCs) was performed with the FACS Canto II flow cytometer (BD Bioscience; 488 excitation laser and 530/30 BP analysis filter) and Flow Logic software V8 (Inivai Technologies, Mentone, Australia) using previously described laboratory protocols [30]. Dichlorofluorescein diacetate (DCF-DA) was used to determine H_2_O_2_ and peroxynitrite (ONOO-) content, and dihydroethidium (DHE) was used to determine superoxide (O2-) content in PBMCs (lymphocytes). Phorbol 12-myristate 13-acetate (PMA) was added to each sample to stimulate ROS production. Data are expressed as fold change of DCF fluorescence units with respect to control.

### 2.9. Statistical Analysis

All results are reported as arithmetic mean and standard deviation (SD). Preliminary results of 10 subjects were considered in the sample size calculation. The calculated sample size was 14 per group to detect differences in primary outcomes reported in this study (e.g., LDF measurement), with a significance level of 0.05 and statistical power of 80% for the paired *t*-test. The Kolmogorov–Smirnov normality test was used to assess the normality of the data distribution. To assess differences within groups (measurements before and after each dietary protocol), the paired *t*-test was used. To assess differences between groups at baseline, the Student’s *t*-test was used. Differences between groups in post-intervention measurements were tested using analysis of covariance (ANCOVA) with baseline (pre-measurement) as a covariate. *p* < 0.05 was considered statistically significant. SigmaPlot, version 11.2 (Systat Software, Inc., Chicago, IL, USA) was used for statistical analysis.

## 3. Results

Table 1 lists the initial anthropometric, hemodynamic, and biochemical characteristics of the subjects. All subjects were lean and normotensive and had normal complete blood counts, serum electrolytes, renal function, hsCRP, fasting blood glucose, and lipid levels. There were no significant differences in any of the measured parameters (e.g., age, BMI, HR, BP, and biochemical parameters) between the athletes in the control and Nutri4 groups at the time of entry into the study protocol. The dietary protocol was completed by all participants.

### 3.1. Anthropometric, Cardiovascular, and Biochemical Parameters

The effects of 3 weeks of consumption of normal (control group) and enriched chicken eggs (Nutri4 group) on anthropometric, hemodynamic, and biochemical parameters are shown in Table 1. Mean arterial pressure (MAP) and serum potassium concentration were significantly decreased after the dietary protocol in the Nutri4 group compared with baseline values. An increase in serum sodium concentration was noted after 3 weeks of consumption of normal chicken eggs. Platelet and fasting glucose levels were higher in the Nutri4 group after the dietary protocol than in the control group. However, all significant changes observed were within the population reference range, so they were not physiologically significant. No significant difference was observed in other anthropometric (BMI, WHR), hemodynamic (systolic BP, diastolic BP, HR), and biochemical (full blood count, urea, creatinine, hsCRP) parameters, as well as serum lipid profile (total cholesterol, triglycerides, LDL cholesterol, and HDL cholesterol) after consumption of Nutri4 or regular chicken eggs compared to initial measurements within the Nutri4 or control group, or when these values were compared between groups.

### 3.2. Serum Fatty Acid Profile, Vitamin E, and Selenium Level Analysis

Serum samples were analyzed for a total of 37 free fatty acids, and only the fatty acids whose concentrations were above the limit of quantitation are listed in Table 2. The serum concentration of cis-4,7,10,13,16,19-docosahexaenoic acid (DHA) increased significantly, and the concentration of palmitic acid (C16:0) decreased significantly after consumption of Nutri4 eggs compared with the first measurement in the Nutri4 group. Serum concentrations of the other free fatty acids measured were similar before and after each dietary protocol in the Nutri4 group. Overall, consumption of Nutri4 eggs significantly decreased the serum n-6/n-3 ratio by approximately 36%. Serum concentrations of measured free fatty acids and the n6/n3 ratio remained unchanged (12% decrease) after regular egg consumption compared with baseline measurements in the control group. Serum C15:0 pentadecyl and C16:0 palmitic acid concentrations and total n6/n3 ratio were significantly lower in the Nutri4 group than in the controls following completion of the corresponding dietary protocol (adjusted for baseline values) (Table 2).

Serum concentrations of Se and vitamin E increased significantly in the Nutri4 group after the dietary protocol, whereas they remained unchanged in the control groups. Serum concentrations of Se and vitamin E were also significantly higher in the Nutri4 group compared with the control group after each nutritional protocol (adjusted for baseline). Serum lutein concentrations before and after the nutritional protocol did not change significantly in any group. These results are shown in Table 3.

### 3.3. Endothelium-Dependent and Endothelium-Independent Vasodilation in Forearm Skin Microcirculation

**Figure 1:**
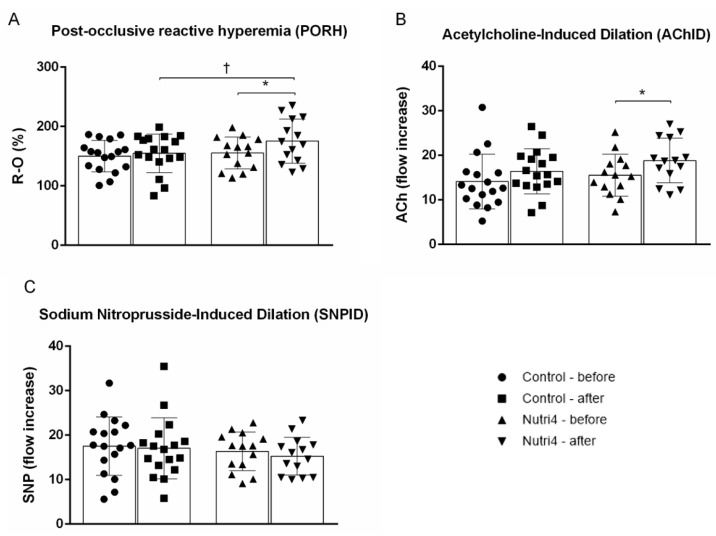
Microvascular endothelium-dependent and endothelium-independent vasodilation of the skin in the control and Nutri4 groups of competitive male athletes. (**A**). Post-occlusive reactive hyperemia, (**B**). Acetylcholine-induced dilation (AChID), and (**C**). Sodium nitroprusside-induced dilation (SNPID). PORH measurement is expressed as the difference between the percentage of flow change during reperfusion and occlusion relative to baseline (R-O%). AChID and SNPID are expressed as the increase in flow after ACh or SNP administration relative to baseline. Control N = 17, Nutri4 N = 14. Results are expressed as arithmetic mean and standard deviation (SD). * *p* < 0.05 before vs. after in Nutri4 group (paired *t*-test); † *p* < 0.05 after control vs. after Nutri4 group—analysis of covariance (ANCOVA) with baseline as covariate.

Both post-occlusive reactive hyperemia (PORH) (Figure 1A) and ACh-induced dilation (AChID) (Figure 1B) of forearm skin microcirculation were significantly increased after consumption of enriched chicken eggs compared with baseline measurements. Consumption of normal chicken eggs resulted in no significant change in PORH and AChID compared with baseline in the control group (Figure 1A,B). PORH was significantly increased in the Nutri4 group compared with controls after the appropriate dietary protocol (adjusted for baseline). SNP-induced dilation (SNPID) was not significantly affected by consumption of either enriched or normal chicken eggs, and it did not differ between the Nutri4 group and controls after the corresponding dietary protocol (adjusted for baseline) (Figure 1C).

### 3.4. Acute Strenuous Training Session and Microvascular Responsiveness Range

**Figure 2:**
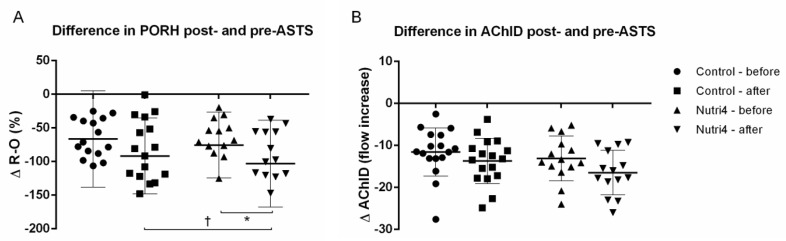
Range of skin microvascular responsiveness to acute strenuous training session (ASTS) in control and Nutri4 groups of competitive male athletes. (**A**). Difference in PORH responsiveness after and before ASTS, ΔPORH = PORH after ASTS—PORH before ASTS; and (**B**). Difference in AChID responsiveness after and before ASTS, ΔAChID = AChID after ASTS—AChID before ASTS. PORH measurement is expressed as the difference between the percent change in flow during reperfusion and occlusion relative to baseline (R-O%), and ΔPORH indicates the difference between the PORH value measured immediately after (post-) and before (pre-) an acute strenuous training session (ASTS). AChID is expressed as the increase in flow after ACh administration compared with baseline flow, and ΔAChID represents the difference in AChID value measured immediately after (post-) and before (pre-) an acute strenuous training session (ASTS). Control N = 17, Nutri4 N = 14. Results are expressed as arithmetic mean and standard deviation (SD). PORH: post-occlusive reactive hyperemia; AChID: acetylcholine-induced dilation; ASTS: acute strenuous training session. * *p* < 0.05 before vs. after within Nutri4 group (paired *t*-test); † *p* < 0.05 after control vs. after Nutri4 group—analysis of covariance (ANCOVA) with baseline as covariate.

Consumption of enriched chicken eggs significantly increased the range of ΔPORH responsiveness to ASTS (Figure 2A), whereas ΔAChID responsiveness to ASTS (Figure 2B) was not significantly altered in the Nutri4 group compared with baseline measurements. No significant change in ΔPORH (Figure 2A) or ΔAChID (Figure 2B) was observed in the control group after the corresponding dietary protocol. The increase in ΔPORH, but not in ΔAChID, was significantly greater in the Nutri4 group compared with the control group after the respective dietary protocol (adjusted for baseline).

### 3.5. Biomarkers of Oxidative Stress and Antioxidant Defense

Myeloperoxidase (MPO) serum protein concentration (MPO pg/mL control before 9719 ± 8623 vs. after 12,019 ± 8316, *p* = 0.233; Nutri4 before 6940 ± 5514 vs. after 11,293 ± 8973, *p* = 0.199) and serum enzyme activity (MPO pmol/mL control before 0.200 ± 0.174 vs. after 0.201 vs. 0.205, *p* = 0.971; Nutri4 before 0.205 ± 0.127 vs. after 0.131 ± 0.089, *p* = 0.086) remained unchanged before and after the respective dietary protocol in both the control and Nutri4 groups. Similarly, serum MPO protein concentration and serum enzyme activity did not differ between the Nutri4 group and controls after the respective dietary protocol (adjusted for baseline).

Serum antioxidant enzyme activity CAT (CAT U/mg protein control before 3.185 ± 0.758 vs. after 3.078 ± 0.808, *p* = 0.681; Nutri4 before 2.440 ± 0.826 vs. after 2.510 ± 0.725, *p* = 0.823), GPx (GPx U/mg protein control before 0.007 ± 0.004 vs. after 0.011 ± 0.006, *p* = 0.088; Nutri4 before 0.013 ± 0.007 vs. after 0.010 ± 0.003, *p* = 0.246) and SOD (SOD U/mg protein control before 9.811 ± 1.109 vs. after 10.061 ± 0.599, *p* = 0.426; Nutri4 before 9.562 ± 0.980 vs. after 10.279 ± 0.884, *p* = 0.061) have not been significantly changed following any of the dietary protocols. In addition, there was no significant difference in the serum activity of the respective enzymes after the dietary protocols (adjusted for baseline) between the control and Nutri4 groups.

**Figure 3:**
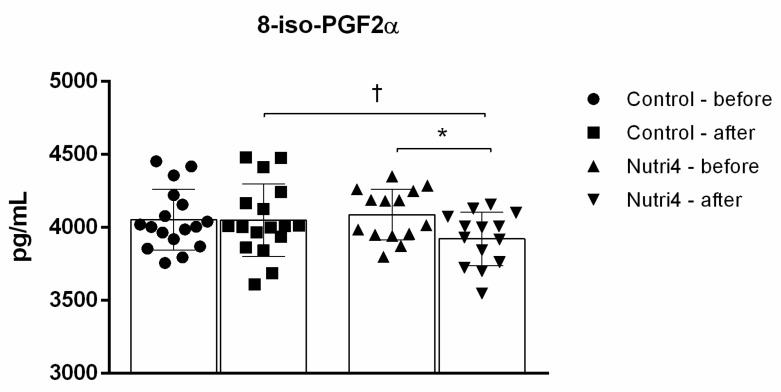
8-iso prostaglandin F2α (8-iso-PGF2α) serum protein concentration in control and Nutri4 groups of competitive male athletes. Control N = 17, Nutri4 N = 14. Results are expressed as arithmetic mean and standard deviation (SD). * *p* < 0.05 before vs. after in the Nutri4 group (paired *t*-test). † *p* < 0.05 after control vs. after Nutri4 group—analysis of covariance (ANCOVA) with baseline as covariate.

The 8-iso-PGF2α serum protein concentration decreased significantly after the three-week protocol in the Nutri4 group, whereas it remained unchanged in the control group (Figure 3). The serum protein concentration of 8-iso-PGF2α was also significantly decreased in the Nutri4 group compared with the control group after each dietary protocol (adjusted for baseline) (Figure 3).

**Figure 4:**
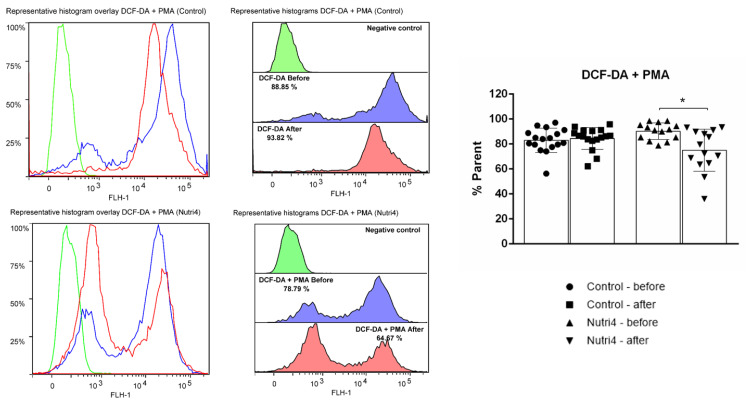
Formation of hydrogen peroxide and peroxynitrite (DCF-DA) in peripheral blood mononuclear cells (PBMCs) in control and Nutri4 groups of competitive male athletes. Control N = 17, Nutri4 N = 14. Results are expressed as arithmetic mean and standard deviation (SD). PMA: phorbol 12-myristate 13-acetate. * *p* < 0.05 before vs. after in the Nutri4 group (paired *t*-test).

Consumption of enriched chicken eggs significantly decreased the formation of hydrogen peroxide and peroxynitrite in peripheral blood mononuclear cells (PBMCs). Consumption of regular chicken eggs did not have any significant effect on the formation of hydrogen peroxide and peroxynitrite in PBMCs (Figure 4).

**Figure 5:**
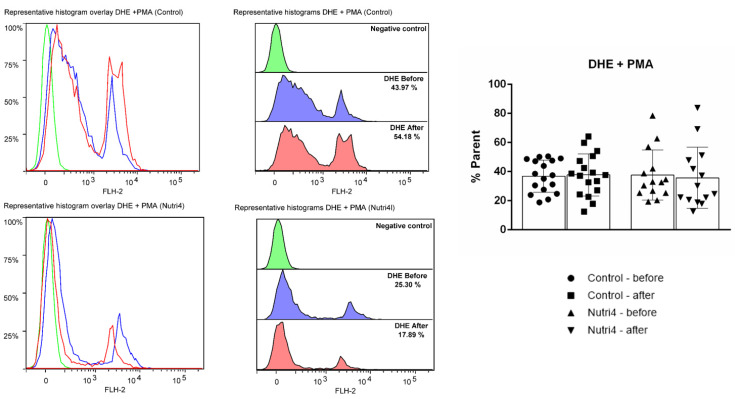
Formation of superoxide (DHE) in peripheral blood mononuclear cells (PBMCs) in control and Nutri4 group of competitive male athletes. Control N = 17, Nutri4 N = 14. Results are expressed as arithmetic mean and standard deviation (SD). PMA: phorbol 12-myristate 13-acetate.

The dietary protocol in both groups did not have any significant effect on superoxide formation in PBMCs in competitive athletes (Figure 5), nor did it differ between groups following respective dietary protocols (adjusted for baseline).

## 4. Discussion

This randomized interventional study is the first one to investigate whether the combined supplementation of four different micronutrients (n-3 PUFAs, Se, lutein, and vitamin E) in the form of functional food (i.e., naturally enriched chicken eggs) has a beneficial effect on endothelial function and oxidative stress levels and whether it increases the capacity of the microvasculature to accommodate for acute exertional stress in a population of competitive male athletes. The most important finding of this study is the significantly improved endothelium-dependent vasodilation of forearm skin microcirculation following consumption of Nutri4 eggs. Importantly, forearm skin microvascular adaptation to the acute exercise stress challenge also increased after consumption of functionally enriched eggs. Another important finding, in addition to functional vascular changes, is a significant decrease in serum protein concentration of 8-isoPGF2α and a decrease in the formation of hydrogen peroxide and peroxynitrite in PBMCs, which suggest a reduction in oxidative stress levels following consumption of the Nutri4 eggs in competitive athletes. Finally, the present study has demonstrated that, while consumption of Nutri4 eggs resulted in increased nutrient (n-3 PUFAs, Se, and vitamin E) serum levels, serum lipid profile and BP remained within the reference range, and no noxious effects have been observed following consumption of fairly large amounts of regular chicken eggs. This is in agreement with our previous studies [5,20] and supports the conclusion that eggs could be safely consumed, particularly in young, nutritionally demanding populations such as athletes.

Approximately 85% of elite track and field athletes use supplements, mostly vitamins and antioxidants, followed by minerals, proteins, creatine, and ergogenic supplements [9]. To avoid taking large amounts of supplements and to achieve balanced nutrition necessary to minimize high-intensity training damage and to improve sports performance, the benefits of functional food in athletes have been intensively investigated [8,9]. Results of available studies suggest that functional food in athlete populations has resulted in endurance improvement, maintained immunity, reduced oxidative stress, and decreased muscle pain; e.g., a study in recreational runners reported these effects after 14 days of consuming date seed powder. Muscle size, strength, and endurance are important to professional athletes, and the role that skeletal muscle microcirculation plays in muscle health is extensive. Microcirculation in skeletal muscle serves to supply oxygen and nutrients but also to remove waste products and heat from skeletal muscle cells [31]. This role is even more pronounced during exercise, as the metabolic rate of muscle can increase up to 50-fold [31]. Therefore, endothelial dysfunction and capillary thinning contribute to exercise intolerance and muscle wasting [31]. The present study demonstrated the enhancement of microvascular endothelium-dependent reactivity after 3-week consumption of Nutri4 eggs in competitive athletes. These results are consistent with the observation that peripheral microvascular response to vascular occlusion (PORH) and ACh were increased in athletes after consumption of chicken eggs enriched with n-3 PUFAs [16]. Long-term regular exercise has a positive effect on vascular function, leading to improved macrovascular and microvascular endothelium-dependent vasodilation compared with sedentary subjects [32,33]. However, the effects of acute strenuous exercise on vascular function are less well studied, particularly with respect to microvascular function. Interestingly, some studies have shown reduced endothelium-dependent flow-mediated dilation (FMD) of the brachial artery after acute exercise [34,35], while others yielded opposite results, reporting improvement of vascular reactivity of large conductance arteries following an acute exercise session. Earlier results from our research group demonstrated that athletes exhibited enhanced baseline microvascular vasodilation compared to sedentary individuals but also that the reduction in endothelium-dependent vasodilation after ASTS was greater in athletes than in sedentary individuals [16]. Moreover, the decrease in PORH and AChID immediately after the ASTS was even more pronounced at the end of 3 weeks of consumption of chicken eggs enriched with n-3 PUFAs [16], which may be in contradiction with the expected results. However, the greater reduction in endothelium-dependent vasodilation in athletes compared with sedentary subjects may indicate increased microvascular responsiveness in the ASTS area or better utilization of vasodilatory capacity, consistent with the hormesis hypothesis [36]. According to this hypothesis, temporary reductions in the endothelial response after ASTS should lead to better long-term endothelial function in athletes, which is also demonstrated in the present study. The potential underlying mechanism for the observed enhanced microvascular reactivity and increased range of microvascular responsiveness to ASTS could be explained by changes in oxidative stress levels that have been observed in the present study due to consumption of functional food enriched in antioxidants.

In the present study, oxidative stress biomarkers (i.e., serum protein concentration of 8-iso-PGF2α and hydrogen peroxide and peroxynitrite formation in PBMCs) were significantly decreased after consumption of nutrient-enriched chicken eggs. Most of the available data on supplementation of nutrients with antioxidant properties (including n-3 PUFAs, Se, and vitamin E) indicate their beneficial effect in reducing oxidative stress levels in a population of athletes. For example, one week of n-3 PUFA supplementation ameliorated the rise in oxidative stress markers after acute resistance exercise in young athletes [37]. Also, 2-month diet supplementation with docosahexaenoic acid (DHA) increased antioxidant capabilities and reduced mitochondrial ROS production in football players [38]. Furthermore, supplementation of vitamins E and C for 7 days reduced oxidative stress after an exercise-induced oxidative stress protocol in football players [39]. And while available studies indicate that vitamin E supplementation appears to be effective at decreasing markers of exercise-induced oxidative stress, evidence of its effects on endurance performance is still lacking [40]. The combination of antioxidant microelements such as DHA and vitamin E in the form of a fortified drink has been shown to have a protective effect against oxidative damage and to increase gene expression of antioxidant enzymes in PBMCs after exercise [41]. Similarly, three weeks of treatment with 100 mcg/day of Se has shown significant changes in the peroxides and glucose-6-phosphate dehydrogenases in eighteen elite athletes, suggesting an antioxidant effect of this element [42]. A different antioxidant compound containing Se, vitamin E, glutathione, and cysteine in the form of pills has also shown an antioxidant effect in fifteen road cyclists after 3 weeks of treatment [42]. Optimal Se plasma levels have been shown to be important in minimizing chronic exercise-induced oxidative effects and modulating the exercise effect on mitochondrial changes [24]. On the other hand, enzymatic activity of CAT, GPx, and SOD remained similar after dietary protocols in both Nutri4 and controls, while MPO enzymatic activity had a tendency to decrease in the Nutri4 group after the protocol; however, that was not significant. Interestingly, it is well accepted that repeated exposure of the organism to low-grade oxidative stress from chronic exercise training leads to an upregulation in the body’s antioxidant defense system, thus providing better protection from oxidative stress during subsequent training sessions [43]. Since both of our examined groups were athletes, this effect of chronic exercise training might be a plausible explanation for the observed lack of significant change in antioxidant defense but decreased oxidative stress biomarkers after an enriched chicken egg dietary regime.

## 5. Conclusions

The present study showed that consumption of chicken eggs functionally enriched with n-3 PUFAs, Se, lutein, and vitamin E resulted in improved endothelium-dependent microvascular reactivity in competitive athletes and an increased range of microvascular responsiveness to ASTS. This is the first study that investigated the combined effects of four nutrients in the form of functional food, and besides improvement in microvascular endothelial function, it demonstrated its effect on a decrease in oxidative stress levels in competitive athletes. Importantly, microvascular adaptation to the acute exercise stress challenge was improved after consumption of Nutri4 eggs. These beneficial pro-endothelial and antioxidant effects of enriched eggs occurred without changes in serum lipids and blood pressure levels, indicating the safety of consuming a large amount of chicken eggs in the population of young athletes. The results of this study could encourage professional athletes to enrich their diet with natural sources of micronutrients in the form of functional foods.

## Figures and Tables

**Table 1:** Anthropometric, hemodynamic, and biochemical parameters in control and Nutri4 group of competitive male athletes.

Parameter	Control		Nutri4	
	Before	After	Before	After
N	17		14	
Age (years)	22 ± 3		23 ± 4	
BMI (kg/m^2^)	24.9 ± 4.0	24.7 ± 3.9	23.6 ± 2.4	23.5 ± 2.4
WHR	0.83 ± 0.09	0.84 ± 0.08	0.83 ± 0.04	0.82 ± 0.04
SBP (mmHg)	122.1 ± 12.2	119.4 ± 12.8	124.1 ± 14.6	118.4 ± 12.0
DBP (mmHg)	70.6 ± 9.2	70.8 ± 8.7	71.0 ± 11.2	70.4 ± 10.8
MAP (mmHg)	87.2 ± 9.4	87.4 ± 9.0	88.8 ± 10.7	85.0 ± 9.1 *
HR (beats per minute)	72.2 ± 9.3	73.8 ± 11.6	70.1 ± 8.9	69.4 ± 11.7
erythrocytes (×10^12^/L)	5.0 ± 0.3	5.0 ± 0.4	5.1 ± 0.3	5.1 ± 0.3
hemoglobin (g/L)	149.1 ± 8.2	148.4 ± 10.6	147.4 ± 7.4	148.3 ± 9.0
hematocrit (%)	42.8 ± 2.4	42.6 ± 2.9	42.6 ± 2.0	43.2 ± 2.2
leukocytes (×10^9^/L)	5.8 ± 1.0	5.7 ± 1.0	6.0 ± 1.1	6.0 ± 1.8
thrombocytes (×10^9^/L)	214.1 ± 57.8	214.8 ± 43.4	233.8 ± 36.4	230.2 ± 61.6 †
urea (mmol/L)	6.2 ± 1.2	6.8 ± 1.3	6.4 ± 0.9	6.3 ± 0.9
creatinine (µmol/L)	91.4 ± 9.9	91.8 ± 9.7	91.1 ± 10.0	90.4 ± 9.2
sodium (mmol/L)	139.4 ± 1.2	140.6 ± 1.9 *	139.8 ± 1.4	139.8 ± 2.1
potassium (mmol/L)	4.2 ± 0.3	4.1 ± 0.2	4.3 ± 0.3	4.1 ± 0.3 *
calcium (mmol/L)	2.6 ± 0.5	2.5 ± 0.4	2.4 ± 0.1	2.4 ± 0.1
iron (μmol/L)	17.0 ± 5.6	17.6 ± 4.6	19.2 ± 9.0	16.1 ± 7.0
transferrin (g/L)	2.5 ± 0.3	2.5 ± 0.3	2.6 ± 0.4	2.6 ± 0.3
glucose (mmol/L)	5.0 ± 0.5	4.8 ± 0.9	5.2 ± 1.0	5.2 ± 1.2 †
hsCRP (mg/L)	0.9 ± 0.8	0.9 ± 1.3	0.7 ± 0.6	0.9 ± 0.8
cholesterol (mmol/L)	4.0 ± 0.7	4.2 ± 0.8	4.4 ± 1.1	4.6 ± 1.1
triglycerides (mmol/L)	1.0 ± 0.5	1.3 ± 1.1	0.9 ± 0.5	1.1 ± 0.7
HDL cholesterol (mmol/L)	1.3 ± 0.3	1.3 ± 0.3	1.5 ± 0.3	1.4 ± 0.3
LDL cholesterol (mmol/L)	2.4 ± 0.6	2.6 ± 0.6	2.6 ± 0.8	2.7 ± 0.8

Data are presented as mean ± standard deviation (SD). N: number of participants; W: women; M: men; BMI: body mass index; WHR: waist-to-hip ratio; SBP: systolic blood pressure; DBP: diastolic blood pressure; MAP: mean arterial pressure; HR: heart rate; hsCRP: high-sensitivity C-reactive protein; HDL: high-density lipoprotein; LDL: low-density lipoprotein. * *p* < 0.05 before vs. after within the group (control or Nutri4)—paired *t*-test. † *p* < 0.05 after control vs. after Nutri4 group—analysis of covariance (ANCOVA) with the baseline value as the covariate.

**Table 2:** Serum fatty acid profile in control and Nutri4 group of competitive male athletes.

Parameter		Control		Nutri4	
		Before	After	Before	After
SFA (μmol/L)					
	C8:0 Caprylic acid	N/F	96.9	29.5	63.8 ± 34.8
	C12:0 Lauric acid	27.2 ± 0.0	38.8 ± 8.6	36.7 ± 21.8	51.2 ± 34.0
	C14:0 Myristic acid	79.8 ± 33.1	106.6 ± 30.8	21.6 ± 23.7	70.7 ± 34.7
	C15:0 Pentadecylic acid	17.8 ± 6.3	21.0 ± 2.7	17.8 ± 2.2	16.7 ± 2.9 †
	C16:0 Palmitic Acid	1701.5 ± 330.2	1872.8 ± 187.6	1811.1 ± 131.2	1634.4 ± 185.0 *†
	C17:0 Margaric acid	21.5 ± 5.0	23.0 ± 1.9	23.0 ± 2.4	20.7 ± 3.6
	C18:0 Stearic acid	808.7 ± 116.6	802.3 ± 73.1	817.2 ± 94.0	772.1 ± 90.3
**PUFA (μmol/L)**					
n-5	C14:1[cis-9] Myristoleic acid	<LOQ	11.07 ± 0.0	<LOQ	11.26 ± 0.0
n-7	C16:1[cis-9] Palmitoleic acid	87.8 ± 36.4	159.3 ± 106.1	84.7 ± 27.5	70.2 ± 16.9
	C17:1[cis-10] cis-10-Heptadecenoic acid	23.0 ± 7.8	20.4 ± 6.4	22.5 ± 8.0	20.0 ± 2.9
n-9	C18:1[trans-9] Elaidic acid	1021.7 ± 307.6	1167.2 ± 0.0	941.2 ± 51.4	697.2 ± 0.0
	C18:1[cis-9] Oleic acid	610.8 ± 595.7	1115.2 ± 694.1	494.6 ± 622.9	1047.4 ± 497.0
	C20:1[cis-11] 11-Eicosenoic acid	15.7 ± 3.3	16.5 ± 3.2	16.4 ± 2.0	14.7 ± 2.6
	C24:1[cis-15] Nervonic acid	6.5 ± 0.0	8.1 ± 1.0	6.9 ± 0.0	<LOQ
n-6	C18:2[cis-9,12] Linoleic acid	1696.2 ± 343.8	1921.4 ± 490.5	1881.5 ± 543.2	1969.4 ± 486.9
	C18:3[cis-6,9,12] gamma-Linolenic acid	31.3 ± 5.9	46.7 ± 23.6	33.2 ± 16.5	28.4 ± 8.8
	C21:2[cis-11,14] Eicosadienoic acid	15.7 ± 3.3	16.5 ± 3.2	16.4 ± 2.0	14.7 ± 2.6
	C20:3[cis-8,11,14] Dihomo-gamma-linolenic acid	94.3 ± 37.1	136.9 ± 92.3	107.6 ± 30.4	90.5 ± 27.1
	C20:4[cis-5,8,11,14] Arachidonic acid	538.8 ± 67.5	600.1 ± 103.8	578.4 ± 53.9	538.3 ± 88.7
n-3	C18:3[cis-9,12,15] alpha-Linolenic acid	21.0 ± 9.9	24.1 ± 3.6	18.1 ± 4.2	23.0 ± 5.9
	C20:4[cis-5,8,11,14] Eicosa-5,8,11,14,17-pentaenoic acid	22.6 ± 3.8	28.4 ± 2.4	20.4 ± 4.3	22.1 ± 5.1
	C22:6[cis-4,7,10,13,16,19] cis-4,7,10,13,16,19-Docosahexaenoic acid	111.1 ± 89.7	207.4 ± 81.5	108.0 ± 32.7	185.4 ± 86.6 *
n6/n3 PUFAs		25.2 ± 19.4	22.1 ± 22.3	19.5 ± 9.3	12.4 ± 3.2 *†

Results are expressed as mean ± standard deviation (SD). SFA: saturated fatty acids; PUFAs: polyunsaturated fatty acids; <LOQ: below limit of quantification; N/F: not found. * *p* < 0.05 before vs. after within the group (control or Nutri4)—paired *t*-test. † *p* < 0.05 after control vs. after Nutri4 group—analysis of covariance (ANCOVA) with the baseline value as the covariate.

**Table 3:** Selenium, vitamin E, and lutein serum concentration in control and Nutri4 group of competitive male athletes.

Parameter	Control (N = 17)		Nutri4 (N = 14)	
	Before	After	Before	After
Selenium (µg/L)	74.5 ± 13.9	70.1 ± 8.2	76.2 ± 15.0	89.3 ± 9.3 *†
Vitamin E (μg/mL)	7.0 ± 4.2	7.1 ± 4.3	7.6 ± 7.8	10.7 ± 5.3 *†
Lutein (μmol/L)	0.089 ± 0.027	0.105 ± 0.061	0.110 ± 0.0409	0.088 ± 0.038

Data are presented as mean ± standard deviation (SD). * *p* < 0.05 before vs. after within the group (control or Nutri4)—paired *t*-test. † *p* < 0.05 after control vs. after Nutri4 group—analysis of covariance (ANCOVA) with the baseline value as the covariate.

## Data Availability

The data that support the findings of this study are available from the corresponding author upon reasonable request.
